# Joint engagement and early language abilities in young children with Down syndrome

**DOI:** 10.3389/fpsyg.2023.1152559

**Published:** 2023-04-28

**Authors:** Laura J. Mattie, Daniela Fanta

**Affiliations:** Department of Speech and Hearing Science, University of Illinois at Urbana-Champaign, Champaign, IL, United States

**Keywords:** Down syndrome, joint engagement, language development, caregiver-child interaction, joint attention

## Abstract

**Introduction:**

Early social strengths likely serve as a foundation for language acquisition for young children with Down syndrome (DS). One way to characterize early social skills is to examine a child’s engagement with a caregiver around an object of interest. The current study examines joint engagement in young children with DS and its relation to language abilities at two-time points in early development.

**Methods:**

Participants were 16 young children with DS and their mothers. At two time points, mother–child free plays were completed and coded for joint engagement. Language abilities were measured at both time points using the Vineland Adaptive Behavior Scales 3rd edition and the number of words understood and produced on the MacArthur-Bates Communication Development Inventory.

**Results:**

Young children with DS spent more time in supported joint engagement than coordinated joint engagement at both time points. Using a weighted joint engagement variable, children with DS who had higher weighted joint engagement had lower expressive language raw scores on the Vineland when controlling for age at Time 1. At Time 2, children with DS who had higher weighted joint engagement had higher expressive and receptive language raw scores on the Vineland when controlling for age. Predictively, children with DS who had a higher weighted joint engagement at Time 1 had a lower number of words produced at Time 2 when controlling for age at Time 1.

**Discussion:**

Our results suggest that young children with DS may compensate for their difficulties with language by using joint engagement. These results highlight the importance of teaching parents to be responsive during interactions with their child to move them into both supported and coordinated engagement, which in turn may foster language development.

## 1. Introduction

Early social skills likely serve as a foundation for language acquisition for young children with Down syndrome (DS). While early social strengths are a hallmark feature of DS [e.g., social orienting, directing eye gaze, vocalizations, gestures, social engagement, and empathy; (Fidler, 2005, 2006; Fidler et al., 2008; Hahn, 2016)], there has been little research examining the relationship between early social skills and language development in this population. One way to characterize early social skills is to examine a child’s engagement with people, objects, and events during a naturalistic interaction (Bakeman and Adamson, 1984; Adamson and Bakeman, 1991; Adamson and Chance, 1998; Adamson et al., 2004). Joint engagement is used to describe periods or episodes of joint attention (Bakeman and Adamson, 1984; Adamson and Chance, 1998; Adamson et al., 2004). That is, joint engagement usually describes the quality of an interaction for a duration of time (Adamson and Bakeman, 1991). In contrast, joint attention is often described, especially as measured in the extant literature, as a point estimate or count that is then used to calculate a rate of joint attention. Joint engagement adds more nuance to joint attention because it differentiates the emergence of joint attention, referred to as supported joint engagement, and the consolidation of skills needed to participate in joint attention interactions with another person, referred to as coordinated joint engagement (Hahn, 2016). Because joint engagement is the foundation for later language development (Smith et al., 1988; Bopp and Mirenda, 2011) understanding this relationship has implications for early language intervention targets and timing. Thus, the purpose of the present study was to examine joint engagement and its relation to language abilities in young children with DS across two-time points.

Joint engagement emerges when a child begins to include a caregiver in their interaction with an object. Before this, children’s engagement, or active attention, is more solely focused on either a person or an object (Adamson and Bakeman, 1991). But as their attention and shifting abilities increase, they begin to share their interests with another person. Because children are learning to consolidate the skills needed to actively share attention (i.e., using eye gaze, affect, gestures, and vocalizations to indicate their attention and interest; Adamson and Bakeman, 1991; Adamson and Chance, 1998), joint engagement is often divided into two forms: supported joint engagement and coordinated joint engagement. Instances of supported joint engagement occur when the child and the caregiver are engaged with the same object, but the caregiver is scaffolding the interaction (i.e., gesturing toward the object or talking about the object) while the child may not consistently reciprocally respond to the caregiver because they are still learning to share their attention (Bakeman and Adamson, 1984; Adamson and Bakeman, 1991; Adamson et al., 2004). Thus, supported joint engagement represents the emergence of the ability to share attention (i.e., joint attention) during which the child needs support from their caregiver to sustain the interaction. This support leads to the ability to share their attention actively and reciprocally with a caregiver (i.e., to use joint attention effectively). Therefore, coordinated joint engagement occurs when the child has gained the skills to share attention and actively engage with the object and caregiver in a dynamic and reciprocal interaction where they are initiating and responding to the actions of the other person around the object (Bakeman and Adamson, 1984; Adamson et al., 2004). It is important to note that although there is a developmental progression of these different engagement states—person, object, supported, coordinated—children will continue to use early forms of engagement even once skills are consolidated based on the demands of the social situation (see Table 1 for definitions and examples of each type of engagement; Bakeman and Adamson, 1984; Adamson and Chance, 1998; Hahn et al., 2016).

**Table 1:** Definitions and examples of engagement states.

Engagement State	Definition	Example
Unengaged	The child is not interacting with objects or the caregiver.	The child is scanning the room.
Person	The child is exclusively interacting with the caregiver in a face-to-face interaction without involving objects or toys.	The child’s body is positioned toward the caregiver and involved in face-to-face interactions (e.g., peek-a-boo, patty cake, etc.).
Object	The child is playing with an object alone, interacting only with the object, and not interacting with or including the caregiver in their play.	The child is focused on building a tower by staking a set of blocks.
Supported Joint Engagement	The child and the caregiver are interacting with the same object, but the child is not actively acknowledging or responding to the caregiver’s participation.	The child and the caregiver are playing with a shape sorter, and the mother encourages the child by commenting or asking questions about the object such as “Green square,” or “Where does that piece go?” or supports the child’s play by handing the child the shapes to insert in the slots. The child might briefly acknowledge or respond to the caregiver but not consistently or for an extended period.
Coordinated Joint Engagement	The child and the caregiver are interacting with the same object, and the child repeatedly acknowledges and responds to the caregiver’s participation in the interaction between the two of them and the object.	The child and the caregiver are playing with a puzzle and the child points to a puzzle piece of a dog and then looks at the caregiver. The caregiver says, “can you put the dog in?.” The child tries to put the piece in place using eye gaze and facial affect to check in with the caregiver.

Engagement states in neurotypical children emerge over the first 2 years of life. Infants begin to engage with their environment within the first month (Adamson and Chance, 1998; Trevarthen and Aitken, 2001). By the second month, infants begin to participate in face-to-face interactions with their caregiver, which is the emergence of person engagement (Adamson and Chance, 1998; Trevarthen and Aitken, 2001). Periods of person engagement start to decrease between 5 and 6 months as infants begin to focus their attention on objects in their environment (i.e., object engagement; Trevarthen and Hubley, 1978; Adamson and Chance, 1998). At this time infants are not yet able to coordinate their interest in objects with the caregiver. However, as infants learn to consolidate their attention and shifting skills, caregivers join in the infant’s engagement with an object leading to the emergence of supported joint engagement after 6 months (Bakeman and Adamson, 1984; Adamson and Bakeman, 1991; Adamson and Chance, 1998). Initially, most interactions between the infant, caregiver, and an object of interest are prolonged periods of supported joint engagement, but as the infant continues to consolidate their attention and shifting skills moments of coordinated joint engagement begin to emerge (Bakeman and Adamson, 1984; Adamson and Chance, 1998). By 12–13 months infants can engage in sustained periods of coordinated joint engagement and by the middle of the second-year infants can use coordinated joint engagement with ease (Bakeman and Adamson, 1984; Adamson and Chance, 1998).

Although most of the research on joint engagement has been conducted in neurotypical children, there is a small body of research in DS. Broadly, research on joint attention and joint engagement in DS often discusses this as an area of relative strength; however, a recent meta-analysis that included studies of both joint attention and joint engagement indicated that these skills, while not a weakness, appear to be commensurate with developmental level (Hahn et al., 2018). Further, the results of this meta-analysis suggest that joint attention and joint engagement may be a strength relative to other aspects of the DS behavioral phenotype and when compared to those with other neurodevelopmental disabilities. Nonetheless, given the small number of studies on joint engagement in DS, it is difficult to draw conclusions about the pattern of performance across engagement statements and the emergence of these skills. For example, Legerstee and Weintraub (1997) found that compared to mental-age-matched neurotypical peers, 8-to-32-month-olds with DS spent more time in supported joint engagement and less time in object engagement and coordinated joint engagement on average across four-time points. In contrast, another study noted that 30-month-olds with DS spent similar amounts of time in supported joint engagement as 18-month-old neurotypical peers (Adamson et al., 2009). Further, in this study, children with DS also spent less time in object engagement and slightly more time in coordinated joint engagement than neurotypical 18-month-olds (Adamson et al., 2009). However, in another study, 20-to-68-month-olds with DS spent more time in coordinated joint engagement and a similar amount of time in supported joint engagement as compared to neurotypical peers matched on receptive vocabulary (Lewy and Dawson, 1992). In a study of Italian 24-month-olds with DS using a similar, albeit not the same coding scheme as other studies of joint engagement, children spent more time in joint attention (i.e., coordinated joint engagement) and less time in passive attention (i.e., supported joint engagement; Zampini et al., 2015). The variability across these studies demonstrates that the use of these skills is fluid and evolving. That is a child can continue to use a less complex engagement state depending on the demands of the situation and they are in the process of learning to use more complex states more competently and efficiently leading to variability in performance. Importantly, there is evidence that coordinated joint engagement increases with development in DS (Legerstee and Weintraub, 1997; Adamson et al., 2009), but at a slower rate than mental-age matched neurotypical peers (Legerstee and Weintraub, 1997). Again, this highlights that the development of joint engagement is progressing.

Examination of language outcomes related to joint engagement in DS has only focused on the most complex forms of supported and coordinated joint engagement in which the child *also* needs to include spoken communication to comment during the interaction (referred to as symbol-infused supported and coordinated joint engagement; Adamson et al., 2004, 2009). While this suggests that the amount of time in symbol-infused supported and coordinated joint engagement accounts for variance in both expressive and receptive vocabulary scores in 30-month-olds with DS (Adamson et al., 2009), it is still unclear how supported and coordinated joint engagement where the child is *not yet* incorporating spoken communication or symbols supports language abilities in DS. Given the variability in the onset of first words in DS (9–24 months; Martin et al., 2009), moments of supported and coordinated joint engagement may be ideal moments for language learning as evidenced by research on joint engagement and joint attention and language in DS (Zampini et al., 2015; Seager et al., 2018) and the use of joint engagement in language interventions for children with autism (Kasari et al., 2008, 2010, 2012). Thus, the present study seeks to describe engagement states in young children with DS across two-time points. In addition, we examined the relationship between joint engagement and language abilities concurrently and predictively. Our research questions were:

1. What is the pattern of engagement for young children with DS at Time 1 and Time 2?
a. Do young children with DS spend different amounts of time in supported joint engagement than coordinated joint engagement at Time 1 and Time 2?
2. What is the relationship between joint engagement and expressive and receptive language ability concurrently and predictively (i.e., joint engagement at Time 1 to language measures at Time 2)?

## 2. Materials and methods

### 2.1. Participants

Participants were 16 young children with DS (9 males, 7 females) and their mothers (*M* age = 42 years, SD = 5.29, range 20–42 years). At Time 1, children were between 12–30 months, and at Time 2, they were between 21–38 months (see Table 2 for child characteristics). Most children were White (62.5%), 31.3% were more than one race, and 6.3% were Asian. For family income, 31.5% were between $20,000–$50,000, 31.4% were between $50,001–$100,000, 31.3% had incomes of $100,001 or above, and 6.3% choose not to report their family income. Most mothers had attended some college (43.8%), 25% graduated college, and 31.3% had a graduate or professional degree.

**Table 2:** Participant characteristics.

Characteristic	Time 1			Time 2		
	*M*	SD	Range	*M*	SD	Range
Child						
Age in months	19.63	4.65	12–30	28.31	5.07	22–38
Vineland Adaptive Behavior Composite	69.07	10.73	45–81	69.81	10.37	51–89
Vineland receptive language raw score	22.75	11.66	4–52	30.19	10.77	11–53
Vineland expressive language raw score	15.81	7.07	4–32	18.88	8.36	5–32
Number of words understood (CDI)	96.87	95.64	6–327	159.87	107.06	28–318
Number of words produced (CDI)	6.27	7.16	0–25	22.47	23.47	1–93

Participants were drawn from two studies examining early language development in DS (12–24 and 18–30 months, respectively) conducted by the first author (see Table 2 for information about language use). For both studies, participants were recruited from the Midwest and Southern regions of the United States through flyers shared with local DS parent groups and early intervention service providers either through email, social media, or newsletters. All children with DS were reported to have normal or corrected hearing and vision, and English was the primary language spoken in their homes.

### 2.2. Procedures

All study procedures were approved by the Institutional Review Board at the University of Illinois at Urbana-Champaign. As part of a larger assessment battery for each study, children and their mothers completed a 15-min free play with a set of developmentally appropriate toys and were instructed to play as they normally would. For the study of children between 12 to 24 months, toys included stacking rings, a set of sensory balls, board books, a cloth book, rattles, a shape sorter, stacking blocks, and connecting rings. For the study of children 18 to 30 months, toys included stacking boxes, animal figurines, a ball, a rattle, connecting rings, board books, a cloth book, an animal puzzle, a teddy bear, 2 plastic bowls, plastic spoons, and plastic forks. Mothers were administered the Vineland Adaptive Behavior Scales 3rd edition (VABS; Sparrow et al., 2016) and completed the MacArthur-Bates Communicative Development Inventory – Words & Gestures (CDI; Fenson et al., 2006). The COVID-19 pandemic interrupted the study for 18-to-30-month-olds and required a transition from in-person assessment to remote assessment. Before the COVID-19 pandemic, participants were visited in person in their homes (Time 1 n = 11; Time 2 n = 9). Families who either enrolled in this study after March 2020 or were due to be seen for their second timepoint after March 2020 (Time 1 n = 5; Time 2 n = 7), completed the free play and were interviewed with the VABS over Zoom. Independent samples *t*-tests indicated no significant differences between those who participated in-person as compared to those who participated remotely on supported or coordinated joint engagement at either time point. Data were managed in REDCap electronic data capture tools hosted at the University of Illinois (Harris et al., 2009, 2019).

### 2.3. Measures

#### 2.3.1. Joint engagement

Joint engagement was coded based on a coding scheme developed by the first author (Hahn et al., 2016; Mattie and Hadley, 2021) based on the coding scheme developed by Bakeman and Adamson (1984). Joint engagement was coded from video recordings of the 15-min free play at both time points. Mothers were asked to play with their child as they normally would. Table 1 provides the definitions and an example of each engagement state. Codes were based on the *child’s* engagement during the interaction. Therefore, the coder was identifying what/whom the child was engaged with to differentiate supported and coordinated joint engagement based on how they were engaging. A full description of the coding scheme is available in Mattie and Hadley, 2021. See Table 1 for definitions and examples of each engagement state.

Video recordings were digitized and coded using Noldus The Observer XT 14 software (Noldus The Observer XT, 2016). Coders would watch the videos in real-time coding for when an engagement state would start and end. An engagement state was defined as “a period of at least 3 s that is characterized by the child’s active interest in people and in objects and events” (Adamson et al., 2004, p. 1,176). To identify the start and end of an engagement state, coders would look for a breakpoint (Newtson, 1973; Bakeman and Adamson, 1984) in the interaction between the child and the mother. When a breakpoint was noted, coders would rewatch the video until they felt they had accurately identified the breakpoint. They would also check that the engagement state lasted for at least 3 s to ensure that it met the criteria for an engagement state.

##### 2.3.1.1. Coder training and reliability

The first author trained the second author on the coding scheme by explaining the scheme in-depth and providing examples of the behavior to be coded by watching videos together. The second author then served as the primary coder for all videos. Reliability was conducted on 8 randomly assigned videos (25% of the video data), which were coded by the first author. Intraclass correlation coefficients (ICCs; Shrout and Fleiss, 1979) were calculated between the primary and reliability coder for the length of time in each engagement state. For each state, the ICCs were unengaged 0.90, object 0.57, SJE 0.76, and CJE 0.85. The ICC for object engagement is lower than the others, this is due to difficulty identifying if there were 3-s of time of object engagement and separating when the state moved from object engagement into either supported or coordinated joint engagement. That is, children were often interested in interacting with their mother, which resulted in either supported joint engagement, due to the mother joining and scaffolding their engagement with the toy or coordinated joint engagement because the child initiated and maintained an active interaction with their mother and the toy. Person engagement rarely occurred in the present study (see Table 3), with only 7 participants using this state; therefore, an ICC was not calculated.

**Table 3:** Frequency and mean proportion of time spent in each engagement state.

Engagement state	Time 1				Time 2			
	Number of children who engaged in each state	Mean proportion of time	SD	Range	Number of children who engaged in each state	Mean proportion of time	SD	Range
Unengaged	15	0.09	0.11	0.00–0.38	15	0.07	0.06	0.00–0.20
Person	7	0.01	0.02	0.00–0.05	7	0.02	0.05	0.00–0.15
Object	16	0.26	0.12	0.10–0.46	16	0.28	0.13	0.09–0.50
Supported Joint Engagement	16	0.52	0.17	0.19–0.77	16	0.53	0.16	0.21–0.76
Coordinated Joint Engagement	11	0.07	0.06	0.00–0.20	9	0.05	0.05	0.00–0.16
Weighted Joint Engagement	16	0.69	0.24	0.19–1.08	16	0.62	0.19	0.21–0.97

#### 2.3.2. Language abilities

Language abilities were measured at both time points using a functional measure (Vineland Adaptive Behavior Scales 3^rd^ edition, VABS) and a parent-reported count of words understood and words produced (MacArthur-Bates Communication Development Inventory, CDI).

##### 2.3.2.1. Vineland Adaptive Behavior Scales 3rd edition, comprehensive interview

The VABS is a standardized caregiver interview of adaptive functioning across three domains: communication, socialization, and daily living (Sparrow et al., 2016). The present study examined the receptive and expressive communication subdomains at each time point to measure functional communication. Items on the VABS are scored by the interviewer on a 3-point Likert scale describing the individual’s ability to do different functional skills independently (*not yet, sometimes, usually/always*). The VABS has well-established reliability and validity.

##### 2.3.2.2. MacArthur-Bates Communicative Development Inventories-Words and Gestures

The CDI is a standardized caregiver report of early communication that provides a checklist of 396 common words their child understands and/or produces *via* speech (Fenson et al., 2006). Although the CDI is standardized, raw scores are not transformed into standard scores. Therefore, if a caregiver reports their child understands 50 words, this number represents the final score. For the present study, language abilities were measured by the number of words understood (receptive vocabulary) and the number of words produced (expressive vocabulary). The CDI has well-established reliability and validity.

#### 2.3.3. Developmental level

Developmental level was measured using the overall level of adaptive functioning. Adaptive functioning was measured by using the Adaptive Behavior Composite from the VABS (M = 100, SD = 15) (Sparrow et al., 2016).

### 2.4. Data reduction

The data extracted from Noldus the ObserverXT was used to calculate the proportion of time spent in each engagement state in seconds by taking the mean duration each participant spent in each state and dividing it by the total time of the observation. This approach was used because although the average length of the mother–child free play was 15 min (i.e., there were slight variations in the total time of each participant’s observation Time 1: *M* = 913.78 s, SD = 86.88 s; Time 2 = *M* = 928.98 s; SD = 29.86 s).

We also calculated a weighted joint engagement score to indicate each child’s level of joint engagement development. This approach accounts for growth and the increasing complexity of joint engagement behaviors (see Hahn et al., 2016). Similar approaches have been used to examine increases in early communication (Luze et al., 2001; Greenwood et al., 2003) and differentiate levels of play complexity (Thiemann-Bourque et al., 2012). Weighted joint engagement was calculated by rank ordering behavior from less to more complex (i.e., supported joint engagement = 1, coordinated joint engagement = 2); therefore, we multiplied each child’s coordinated joint engagement score by 2 and added the supported joint engagement score of each child (Hahn et al., 2016). For example, if the proportion of time spent in coordinated joint engagement was 0.20 and the proportion of time spent in supported joint engagement was 0.30, then the weighted joint engagement score would be 0.70 (i.e., [0.20 × 2] + 0.30 = 0.70; see Table 3 for means, standard deviations, and ranges).

### 2.5. Data analysis

For our first research question, descriptive statistics were used to explore the pattern of engagement at Time 1 and Time 2. Next, we used paired-sample *t*-tests to examine if there were differences between supported and coordinated joint engagement at each Time 1 and Time 2. The proportion of time spent in each engagement state was used for these analyses.

For our second research question, we used partial correlations controlling for child chronological age to examine the relationship between joint engagement and language abilities concurrently and predictively (receptive and expressive raw scores from the Vineland and the number of words understood and the number of words produced from the CDI). For these analyses, the weighted joint engagement score was used. For the predictive correlations weighted joint engagement at Time 1, controlling for chronological age at Time 1, and language abilities at Time 2 were used.

## 3. Results

### 3.1. Pattern of engagement

Young children with DS, on average, spent the most time in supported joint engagement followed by object engagement with little time spent in the other engagement states, including coordinated joint engagement at both time points (see Table 3). Two children who used coordinated joint engagement at Time 1 did not use coordinated joint engagement at Time 2. In addition, four children with DS never used coordinated joint engagement at either time point.

#### 3.1.1. Differences in supported and coordinated joint engagement

Paired sample *t*-tests, indicated that young children with DS spent significantly more time in supported joint engagement than coordinated joint engagement at each Time 1 (*t*[15] = 8.31, *p* < 0.001, *d* = 2.08) and Time 2 (*t*[15] = 11.96, *p* < 0.001, *d* = 2.99).

### 3.2. Relationship between weighted joint engagement and language abilities

#### 3.2.1. Concurrent relationship between weighted joint engagement and language abilities at Time 1

At Time 1, young children with DS who had higher weighted joint engagement had lower expressive language raw scores on the Vineland at Time 1 when controlling for chronological age, *r* = −0.70, *p* = 0.005. No other significant relationships emerged at Time 1.

#### 3.2.2. Concurrent relationship between weighted joint engagement and language abilities at Time 2

At Time 2, young children with DS who had higher weighted joint engagement had higher expressive and receptive language raw scores on the Vineland when controlling for chronological age (*r* = 0.52, *p* = 0.06; *r* = 0.79, *p* < 0.001). No other significant relationships emerged at Time 2.

#### 3.2.3. Predictive relationship of weighted joint engagement at Time 1 to language abilities at Time 2

Young children with DS who had a higher weighted joint engagement at Time 1 had a lower number of words produced at Time 2 when controlling for chronological age at Time 1, *r* = −0.58, *p* = 0.03. No other significant relationships emerged.

## 4. Discussion

The present study sought to characterize joint engagement in young children with DS and the relationship between joint engagement and language abilities. Our results indicate that children with DS spent more time in supported joint engagement than in any other engagement state. Further, they spent significantly more time in supported than coordinated joint engagement at both time points. This pattern is consistent with patterns observed in neurotypical 12-to-15-month-olds (Bakeman and Adamson, 1984) and 18-month-olds (Adamson et al., 2009), suggesting that children with DS may demonstrate delays in the use of supported and coordinated joint engagement as compared to their chronological age. However, the pattern of more supported than coordinated joint engagement is consistent with one of the previous studies of joint engagement in 12-to-26-month-olds with DS (Legerstee and Weintraub, 1997). Our study also extends this finding, suggesting that this pattern may continue past the child’s 3rd birthday. It is important to note that, Lewy and Dawson (1992) found a pattern of more coordinated joint engagement than neurotypical peers. The age range in this study was quite large, 20–68 months (mean age 37 months). Thus, it is possible that the shift to using more coordinated joint engagement starts after 3 years and increases as children with DS develop. Thus, one important consideration for future research is to examine when children with DS transition to using more coordinated joint engagement than supported joint engagement. This would provide important information about the consolidation of these skills and lead to an increase in reciprocal interactions with others.

In the current study, we used a weighted joint engagement variable to examine the relationship between joint engagement and language abilities at two-time points. At Time 1, children with DS who had higher joint engagement had lower expressive language when controlling for chronological age. This suggests that young children with DS may compensate for their difficulties with expressive language by using joint engagement (Jenkins and Ramruttun, 1998). Research on prelinguistic communication in children with language delays and/or intellectual disabilities has noted similar patterns of using these skills to compensate for expressive language delays (Bishop et al., 2000; LeBarton and Iverson, 2017; Bordenave and McCune, 2021). This is further supported by our finding that children who had higher joint engagement at Time 1 had a lower number of words produced at Time 2. That is, the increased use of joint engagement at Time 1 to compensate for difficulties with expressive language appears to continue into later development (Time 2) as children continue to struggle with expressive language. An alternative explanation for this finding is that caregivers may provide more scaffolding to children with DS who have limited expressive language abilities, leading to an increased frequency of supported joint engagement. This information is particularly important for clinicians to continue to target joint engagement skills, regardless of the strength of these skills in young children with DS, because they are foundational skills that will support later expressive language abilities and outcomes (e.g., Kasari et al., 2008, 2010, 2012).

Nonetheless, it does appear that later in development at Time 2, increased use of joint engagement is associated with higher expressive and receptive language abilities when controlling for chronological age. These findings, that is concurrent relationships at Time 2, align with the view that the dynamic process of joint engagement supports language development (Bakeman and Adamson, 1984; Adamson et al., 2004; Paparella and Kasari, 2004; Bottema-Beutel et al., 2014). Further, the association between joint engagement and receptive and expressive language abilities in DS is echoed in prior research on joint attention—which is similar to joint engagement—and related supportive skills, like triadic eye gaze, (Mundy et al., 1995; Harris et al., 1996; Seager et al., 2018; Hahn et al., 2019). Thus, targeting joint engagement may be especially helpful for language interventions for children with DS into the second year. These interventions may be particularly useful for children with DS who are compensating for expressive language delays with their joint engagement abilities. In addition, it may be especially important to focus on the transition to coordinated joint engagement, which may lead to more salient opportunities for word learning (Mattie and Hadley, 2021) and have a greater impact on language development (Adamson et al., 2004).

Although, the existing interventions that target joint engagement and other prelinguistic communication skills have demonstrated lasting effects on language outcomes for children with intellectual and developmental disabilities and children with language delays (Yoder and Warren, 2002; Fey et al., 2006, 2013; Landry et al., 2008; Kasari et al., 2012), intervention studies that have included children with DS within their samples of children with intellectual and developmental disabilities, have reported mixed findings on their impact for children with DS. For example, Yoder and Warren (2002) found that children with DS had a greater increase in requesting when they were in the no-treatment control group. In contrast, Fey et al. (2006) reported no differences in outcomes if children had DS or not. Nonetheless, our results highlight the importance of teaching caregivers to be responsive to their child’s attention when interacting. For example, if a child is engaged with a cat figurine, a caregiver can join their attentional focus by pointing to the figurine saying, “That’s a cat.” Thus, providing clear linguistic input that can help with word learning (Rowe and Snow, 2020; Mattie and Hadley, 2021). This can also set up an opportunity for the child to respond to the caregiver’s communication; thus providing scaffolding that can support the child in moving into supported and coordinated joint engagement. Continued research on the implementation of early language interventions for young children with DS, including caregiver language input, is needed.

### 4.1. Limitations and future directions

There are several limitations to the present study. First, our sample size is small and a sample of convenience. Although this sample size is not uncommon in DS research, our results should be replicated with larger samples. Also, there was an overlap in chronological ages at Time 1 (12–30 months) and Time 2 (21–38 months). Thus, we were not able to fully explore the ages at which children may start to transition to using coordinated joint engagement with more frequency. In addition, future research should explore if the transition to coordinated joint engagement is facilitated by increased episodes of supported joint engagement with their caregiver at an earlier age. Conducting a more nuanced examination of joint engagement, such as dividing supported joint engagement into lower-order and higher-order skills (Bottema-Beutel et al., 2014) is also needed. Similarly, describing other behaviors associated with joint engagement (i.e., gestures, vocalizations) may also help to elucidate the transition to coordinated joint engagement. It is important to note, that each child had at least 6 months in between their time points and, therefore, reflects changes observed in each child. Therefore, future research is needed to explore age-related changes in joint engagement and its association with language growth.

We also combined data from two pilot studies, as is becoming common practice to achieve larger samples (e.g., shared data repositories), but one of these studies was interrupted by the COVID-19 pandemic leading to a shift in how the data was collected (i.e., *via* Zoom instead of in-person). These different methods may influence how mothers interacted with their child. Although, both involve them being observed by the research team, being at a distance versus physically present in their home may have changed their behavior. However, other than if the family participated remotely, the inclusion criteria and methodology were the same (i.e., instructions for free play, etc.). In addition, the COVID-19 pandemic affected our ability to collect a direct measure of nonverbal cognitive abilities. Future studies should explore the role of nonverbal cognitive abilities on the relationship between joint engagement and language abilities. Person engagement was rarely used in this study. Although, mothers were told to play as they normally would, providing them with a set of toys may have led them to play more with toys than with face-to-face interaction games, like peek-a-boo. We focused our analysis on words produced on the CDI, but this variable does not account for the child’s use of sign language. Sign language is often used by children with Down syndrome as a form of alternative and augmentative communication (AAC; Launonen, 1996; Wright et al., 2013). Future studies should also examine the relationship between joint engagement and expressive language as measured by the use of sign language and other forms of AAC. Lastly, both studies focused on early language development. This may have led to more participation from families who were concerned about their child’s language abilities.

## 5. Conclusion

Joint engagement appears to be an important skill for language development in DS. Together these results highlight the importance of teaching caregivers to be responsive during interactions with their child to move them into both supported and coordinated engagement, which in turn may foster language development. Continuing to explore the early language profile, and skills that support it, in DS can help to identify targets for early language interventions in this population. In addition, exploring this profile can help determine the roots of the language difficulties in later development in DS and support the identification of skills that can be targeted early to promote better language outcomes later in development.

## Data availability statement

The raw data supporting the conclusions of this article will be made available by the authors, without undue reservation.

## Ethics statement

The studies involving human participants were reviewed and approved by University of Illinois at Urbana-Champaign Institutional Review Board. Written informed consent to participate in this study was provided by the participants’ legal guardian/next of kin.

## Author contributions

LM conceptualized the study, performed the analysis, and drafted and edited the manuscript. DF oversaw data coding and assisted with drafting and editing the manuscript. All authors contributed to the article and approved the submitted version.

## Funding

This research was supported by grants from the Campus Research Board grant from the Office of the Vice Chancellor for Research at the University of Illinois Urbana-Champaign (Mattie) and the Center on Health, Aging, and Disability’s Pilot Grant Program at the University of Illinois Urbana-Champaign (Mattie).

## Conflict of interest

The authors declare that the research was conducted in the absence of any commercial or financial relationships that could be construed as a potential conflict of interest.

## Publisher’s note

All claims expressed in this article are solely those of the authors and do not necessarily represent those of their affiliated organizations, or those of the publisher, the editors and the reviewers. Any product that may be evaluated in this article, or claim that may be made by its manufacturer, is not guaranteed or endorsed by the publisher.
